# Editorial: Abiotic stress adaptation and tolerance mechanisms in crop plants

**DOI:** 10.3389/fpls.2023.1278895

**Published:** 2023-09-22

**Authors:** Bhaskar Gupta, Jiban Shrestha

**Affiliations:** ^1^Department of Zoology, Government General Degree College, Singur, West Bengal, India; ^2^Nepal Agricultural Research Council, National Plant Breeding and Genetics Research Centre, Khumaltar, Lalitpur, Nepal

**Keywords:** abiotic stress, drought, omics, polyamine, stress adaptation

A major challenge for world agriculture involves the production of 60% more food crop to feed 9.3 billion people worldwide by 2050 (UN, 2012). In recent years, the escalating threat of climate change and its associated environmental stresses (osmotic stress, drought, UV-B radiation, mineral nutrient deficiency, pH variation, metal toxicity and extreme temperatures like heat waves and cold waves have significantly impacted the growth, survival, longevity, reproduction, and global productivity of crop plants (Redondo-Gómez, 2013). Abiotic stress involves modulations in various physiological and metabolic processes, depending on the severity and duration of the stress, and eventually inhibits crop production. To adapt to these hostile environmental cues, plants have developed various physiological and biochemical mechanisms, which include, but are not limited to; (1) ion homeostasis and compartmentalization; (2) ion transport and uptake; (3) biosynthesis of osmoprotectants and compatible solutes’ (4) activation of antioxidant enzyme and synthesis of antioxidant compounds; (5) synthesis of polyamines; (6) generation of nitric oxide (NO); (7) hormone modulation; and (8) expression of stress regulatory genes (Ghosh and Xu, 2014; Gupta and Huang, 2014; Saha et al., 2015; Hasanuzzaman, 2020; Zhang et al., 2022). These adaptive responses help plants survive and recover from stress, while non-adaptive responses result in damage or dysfunction (Zhang et al., 2022).

The Research Topic titled “*Abiotic stress adaptation and tolerance mechanisms in crop plants*,” explores cutting-edge research on mechanisms that allow crop plants adapt to and tolerate abiotic stresses. This editorial aims to provide an overview and critical analysis of the book’s contents, which include 35 original research articles and 3 review articles, highlighting the key insights and advancements made in this field. The book explores key topics such as molecular responses, genetic aspects, physiological adjustments, biochemical signals and agronomic strategies that enable crops to cope with adverse environmental conditions.

## 1 Transcriptomics, genomics and metabolomics: unveiling pathways and plant stress responses

Transcriptomics and genomics are two fields of study that use different methods and data to understand the molecular biology of plants. Rice (*Oryza sativa* L.) is a major food crop for almost half of the world population. In this Research Topic, Ahmad demonstrated that the identification of numerous important QTL genes, promoter elements, transcription factors and miRNAs involved in rice response to abiotic stress was made possible by a spectrum of genomic and transcriptomic techniques. In fact, multiple “omics” approaches have emerged as successful technologies for plant systems over the last few decades (Yang et al., 2021).

The study by Li et al. focused on analyzing the roots of two different wild soybean seedling ecotypes to understand how they adapt to low-phosphorus (P) stress. The study found that one of the ecotypes, barren-tolerant wild soybean (GS2), enhanced soil P uptake through mechanisms such as increasing root length, promoting the synthesis and secretion of lignin and organic acids, and upregulating certain genes [inorganic phosphate transporter (PHT1-2 and PHT1-3), acid phosphatase (ACP1), and purple acid phosphatase] related to phosphate transport and phosphatase activity. This helped maintain stable P content in plants, and thus better adaptation to low-P stress. Additionally, GS2 showed increased activities of pectin esterase and phospholipase C to reuse structural P, generated sulfoquinovosyl diacylglycerol to maintain biofilm structure stability, and enhanced the synthesis of antioxidants like anthocyanins to reduce oxidative damage due to P deficiency. The study identified specific transcription factors (HSFA6b and MYB61) as key regulators in the resistance of barren-tolerant wild soybeans to low-P stress. Overall, this research offers insights into plant adaptation to different P levels and provides quantitative parameters for resource evaluation.

The comparative transcriptome analysis of drought-tolerant and drought-sensitive genotypes of ‘tartary buckwheat’, under field soil drought stress, was performed by Meng et al., utilizing the weighted gene co-expression network analysis (WGCNA) data mining tools (Langfelder and Horvath, 2008), which revealed key genes and metabolomic pathways involved in tartary buckwheat drought tolerance. Furthermore, Ji et al., through detailed transcriptome analysis, demonstrated the regulation of salt tolerance in the roots of *Zea mays* by L-histidine, one of the standard amino acids in proteins and critical for plant growth and development. Hu et al. did a comprehensive transcriptomic and metabolic regulatory network characterization of drought responses in tobacco, which could pave the way for engineering manipulation for breeding new tobacco varieties with enhanced drought tolerance. The strategy of integrating linkage mapping and comparative transcriptome analysis has been deployed by Geng et al. to discover candidate genes associated with salt tolerance in rice. Wu et al. measured changes in metabolite levels in the roots and needles of pine (*Pinus taeda*) seedlings under long-term drought stress, and identified major metabolic pathways in pine seedlings related to drought stress. This work lays the foundation for understanding the metabolic mechanisms of drought tolerance in coniferous species.

Priming is a strategy to change the performance of seeds to cope with stress. Halopriming applied to seeds triggers plant stress memory, preventing the deleterious impact of abiotic stresses such as drought and salinity (Pagano et al., 2023). In a study on two contrasting Chilean quinoa ecotypes (*Chenopodium quinoa*), Cifuentes et al. showed that metabolic imprint induced by seed halo-priming promotes a differential physiological performance in two contrasting quinoa ecotypes.

Drought and salt stress are serious threats to crop productivity worldwide and identification of genetic, biochemical, and molecular cues for increasing plant adaptation is of paramount significance. Chen et al. demonstrated that purple stem *Brassica napus* plants, in comparison to green stem ones, exhibits higher photosynthetic efficiency, antioxidant potential and anthocyanin biosynthesis - related gene expression against drought stress. Based on their findings, it can be concluded that green stem genotypes are more sensitive to drought stress than their purple stem counterparts. Furthermore, this research article also provides practical guidance for plant biologists who are developing stress-tolerant crops by using anthocyanin biosynthesis or regulatory genes. Betaine aldehyde dehydrogenase (BADH) is a key enzyme in the synthesis of the osmotic regulator betaine in wheat. Overexpression of *TaBADH* (*T. aestivum* L.) in *Arabidopsis* was found to enhance betaine accumulation and salt tolerance (Sun et al., 2019). Yu et al. cloned a region of the *TaBADH-A1* promoter and genomic DNA that included the introns and exons, from four Chinese wheat cultivars. The authors have found seven haplotypes of the *TaBADH-A1* gene in wheat, the seven haplotypes were divided with 254 bp indel into two alleles. The expression level of *BADH-A1b* was shown to be significantly higher than that of *BADH-A1a* under drought and salt stress. *BADHA1b* increased the betaine content of wheat and further enhanced its drought resistance and salt tolerance. *BADH-A1b*, on the contrary, had no effect on the agronomic traits of wheat grown under normal conditions. This study provides evidence indicating that *BADH-A1b* could be an excellent candidate allele for breeding to improve the drought resistance and salt tolerance of wheat. The study of Jiao et al. describes the miRNA–mRNA analysis and molecular mechanisms involved in drought response in the maize root system. Moreover, four metabolic pathways, namely, “plant hormone signal transduction”, “phenylpropane biosynthesis”, “glutathione metabolism”, and “starch and sucrose metabolism”, were predicted to have important roles in the response of the maize root system to drought. Jing et al. conducted field experiments to quantitatively describe the persistent effects of drought on summer maize growth indexes and yield. Furthermore, Jing et al. constructed the comprehensive resilience assessment method based on the growth indexes and quantitatively analyzed the resilience under different drought degrees and durations. Gebre et al. showed that currently cultivated commercial soybean [*Glycine max* (L.) Merr.] cultivars grown in Canada (Ontario) differ significantly in their response to drought stress in terms of their growth, yield and yield components (pod number, seeds per pod, single-seed weight). Lignin is a complex phenolic compound, which is an important component of the plant cell wall. The effects of low-light stress on lignin metabolism, and lignin monomer composition, as well as their relationships with lodging resistance, have been explored by Luo et al.

## 2 Addressing other abiotic stressors

Heavy metal stress causes devastating losses in crop productivity and affects ecology and human health worldwide. Plants try to overcome and adapt to heavy metal toxicity to survive because it adversely affects plant growth, development, reproduction, physiological-biochemical processes, and crop productivity by disturbing cellular ionic balance, metabolic balance, cell membrane integrity, and protein and enzyme activities. The role of ABA in plant abiotic stress signaling and adaptation is well established. Kumar et al. have done an exhaustive review of ABA metabolism, transport, crosstalk with other plant growth regulators, and its role in heavy metal stress mitigation. Toxic metal-induced overaccumulation of anthocyanin in plants can oxidize proteins and break DNA. Here, a study by Zhang et al. presents the latest evidence to update the positive role of exogenous proline in regulating jasmonate signals and activating the MYB-bHLH-WDR complex to repress anthocyanin accumulation in rice plants under Cr(VI) stress. Additionally, Zhu et al. demonstrated that cotton straw biochar and compound *Bacillus* biofertilizer reduce Cd stress on cotton root growth by regulating the antioxidant enzyme system, the pathways of ABC transporters and phenylalanine metabolism and root exudates. The effect of Zn-lysine and biochar (alone and in combination) on the growth and physiology of wheat (*Triticum aestivum* L.) to alleviate salinity stress has been studied comprehensively by Aibdin et al. Their results indicate that Zn-lysine and biochar alone and in combination enhanced the biomass of wheat, improved the antioxidant activity in leaves and assisted the nutrient uptake by wheat plants under salt stress.

The implication of silicon in regulating physiological and biochemical mechanisms of contrasting bread wheat cultivars under terminal drought and heat stress environments has been studied by Ashfaq et al. Their research shows that silicon treatment has the potential to mitigate the detrimental effects of individual and combined stress of drought stress, heat stress, and drought-heat stress at an early grain-filling stage in susceptible and tolerant wheat cultivars in a controlled environment. Another study on silicon, using transcriptomic and metabolomic data reveal that silicon enhances the adaptation of rice under dry cultivation by improving flavonoid biosynthesis, osmoregulation, and photosynthesis (Jiang et al.). The authors claimed that the establishment of the system for increasing the yield of rice under dry cultivation provides theoretical and technical support, thereby promoting the rapid development of rice in Northeast China, and ensure national food security. Autotoxicity is a key factor that leads to obstacles in continuous cropping systems, which refers to the phenomenon where the successive planting of the same crop on the same land leads to a decline in crop yield and quality (Xiao et al., 2019). Lyu et al. show that exogenous silicon alleviates the adverse effects of cinnamic acid-induced autotoxicity stress on cucumber seedling growth. Brassinosteroid hormones multitask to smoothly regulate a wide array of vital physiological and biochemical processes in plants, and have been found to play a significant role is diverse plant abiotic stresses, including salt and alkali stress (Chaudhuri et al., 2022). Sun et al. elaborated on the information available on the main mechanisms underlying plant tolerance to abiotic stress by further analyzing the mechanism of exogenous analog brassinolide in improving the alkaline tolerance of apple plants (*Malus hupehensis*).

The evaluation of irrigation water efficiency plays an important role in the efficient use of agricultural water resources and the sustainable development of agriculture (Dong et al., 2020). Notably, regulated deficit irrigation, a water-saving irrigation method widely adopted in China, can be employed to reduce water consumption without causing a significant wheat yield loss (Sheng et al.). Tejera-Nieves et al. discussed the underlying mechanism and revealed that the seasonal decline in leaf photosynthesis in perennial switchgrass (*Panicum virgatum* L.) can be explained by sink limitations and water deficits.

## 3 Polyamines: growth regulators and stress responders

Polyamines are critical players in growth, developmental processes and stress responses in plants (Gupta et al., 2013; Gupta et al., 2016; Sengupta et al., 2016; Chen et al., 2019). Here, whole genome-wide screening of polyamine oxidase *(PAO)* genes in maize was done by Xi et al. and nine *ZmPAO* genes were identified in this study, named as *ZmPAO1*-*9*. Extensive functional analysis of these *PAO* genes established their essential roles in abiotic stress tolerance (heat, drought, and salinity stress). The symbiotic relationship between soybean (*Glycine max* L.) and rhizobium to form root nodules, which can fix nitrogen from the air for their own growth, is well established (Mulder et al., 2005). Lyu et al., in their research article, have proposed the systemic regulation of soybean (*Glycine max* L.) nodulation and nitrogen fixation by nitrogen via isoflavones. Heat shock proteins (HSPs) are evolutionarily conserved, ancient intracellular molecular chaperones that widely exist in almost all organisms (Dattilo et al., 2015). When organisms are subjected to environmental stresses, HSPs have been shown to improve the adaptation of organisms to stress by stabilizing the cell structure, transporting, and folding auxiliary proteins and maintaining cell function (Gupta and Huang, 2014; Yadav et al., 2021). Huang et al. have done genome-wide analysis of HSP20 gene family in cucumber (*Cucumis sativus* L.) and studied their expression patterns under heat stress. These findings are expected to provide a reference for revealing the role of HSP family members in cucumber development regulation and stress response and serve as molecular resources for further cultivating new varieties that are resistant to high temperatures. The NAC (NAM, ATAF1/2, and CUC2) transcription factor family is one of the largest families of transcription factors in plants. NAC transcription factors are named after no apical meristem (NAM) proteins found in *Petunia* hybrids, *Arabidopsis* transcription activation factor (ATAF1/2) proteins, and cup-shaped cotyledon (CUC2) proteins of *A. thaliana* (Jiang et al., 2021). Wen et al. in their study found that a tobacco *NAC* gene (*NtNAC028)* was rapidly upregulated in response to high salinity, dehydration, and abscisic acid (ABA) stresses, suggesting a vital role of this gene in abiotic stress response. *NtNAC028* loss-of-function tobacco plants generated *via* CRISPR-Cas9 showed delayed leaf senescence and increased tolerance to drought and salt stresses. Further mutation and overexpression analysis revealed that *NtNAC028* might act as a vital regulator for plant stress response likely by mediating ROS scavenging ability. The study by Karwa et al. analyzed the impact of heat stress on rice growth and yield metrics and demonstrated the ameliorating effect of exogenous spermidine application in inducing heat stress tolerance in rice at the reproductive stage.

## 4 Harnessing molecular tools for stress adaptation

The ground-breaking gene editing tool, CRISPR/Cas9 (clustered regularly interspaced short palindromic repeats/CRISPR-associated protein 9), has opened new and exciting avenues for the analysis of gene function. While the original methodology dealt with targeted gene disruption, recent technological research advancements have yielded a wide spectrum of tools to modify genes and gene expression in various ways (Horodecka and Düchler, 2021). Here, Zhong et al. have utilized the CRISPR/Cas9 mediated gene-editing tool to identify and comprehensively analyze the drought tolerance potential of Soybean *(G. max [L.] Merr.) GmHdz4* transcription factor gene. Hu et al. investigated the influence of drought stress on photosynthetic parameters, stomatal status, and chlorophyll content of two different cultivars of an economically and medicinally important plant species, *Zanthoxylum bungeanum*, with contrasting drought tolerance phenotype. The role of rice protein phosphatase 1 regulatory subunits in growth and abscisic acid (ABA) mediated abiotic stress signaling was extensively studied by Jadoon et al. They have established that rice OsINH2 and OsINH3, the homologs of *Arabidopsis* INH2 and INH3 respectively, actively participate in the reproductive growth of rice, regulate ABA-mediated inhibition of seed germination, and contribute to ROS homeostasis. Recently, the research article by Cimini et al. (2022) has highlighted the multifactorial regulation of glutathione metabolism behind salt tolerance in rice.

An integrative analysis of transcriptome and metabolome explored (Yang et al.) the relationship between differentially expressed genes (DEGs) and significantly regulated metabolites (SRMs) under salt stress in two rice cultivars [HD961 (salt-tolerant) and 9311 (salt-sensitive)]. The HD961 cultivar showed enriched glutathione metabolism pathways, with specific SRMs and DEGs. Correlations were found between certain genes [16 genes (12 up-regulated genes and four down-regulated genes)] and metabolites (GSH, dehydroascorbic acid, L-cysteine) involved in glutathione metabolism, highlighting their potential roles in GSH synthesis under salt stress.

## 5 Temperature extremes and their implications for plant growth and productivity

Global warming, unpredictable weather patterns, and extreme temperatures have become more frequent, harnessing ever-increasing environmental stress challenges for plants. Such stress disrupts the normal growth and development of plants and is a major threat for food safety and security (Jagadish et al., 2021). Understanding the primary molecular and physiological mechanisms that drive the adaptation of crop plants to various abiotic stresses is crucial to bridge the knowledge gap in this area. Yang et al. evaluated the effect of high night temperature on the yield and seed compositions of soybean during seed filling stage and analyzed the dynamic changes in photosynthetic physiological characteristics, dry matter accumulation and non-structural carbohydrates linked to stress tolerance triggered by high night temperature. Yang et al. have argued that in the context of global warming, breeding soybean varieties with low respiration and high energy efficiency is one of the most important ways to effectively address the world food crisis. Goswami et al., in their review article, delved into recent insights of physio-biochemical and molecular stress regulators and their crosstalk for low- temperature stress responses in fruit crops. The regulatory function of non-coding small RNAs, especially miRNAs, in tomato plants has been comprehensively explored by Li et al. miRNAs are endogenous non-coding small RNAs that participate in plant development, organ formation, signal transduction, and stress response (Cai et al., 2009). Li et al. constructed small RNA libraries from wild-type tomato plants and ABA-deficient mutant plants under normal growth and low- night temperature stress conditions. They demonstrated that miRNA162 regulates stomatal conductance through regulation of the abscisic acid (ABA) signaling pathway in tomato. To study the genome-wide gene expression under heat, Tang et al. performed a time-course (0.5 to 24 h) transcriptome analysis in the leaf and root of 40-day-old pepper plants under control and 40 °C. Comparative transcriptome analysis between the heat-treated and control plants identified differentially expressed genes, which showed the largest degree of change at 24 h. Interestingly, their work revealed that more differentially expressed genes were identified in the root than in the leaf. Additionally, Tang et al. analyzed the gene expression of 25 heat shock factor genes (HSFs) in pepper after heat stress, identified five of these HSFs that responded to heat stress, and characterized the role of these genes in heat-tolerant and heat-susceptible pepper lines. These findings are expected to improve the knowledge and understanding of the genome-wide heat stress response in pepper plants. High seed quality is a key to agricultural production, which is increasingly affected by climate change. Nagel et al. studied the effects of drought and elevated temperature on key seed quality traits of two malting barley (*Hordeum sativum* L.) genotypes during seed production and demonstrated that drought, but not elevated temperature, affected the metabolism of amino acids, organic acids, osmolytes and nitrogen assimilation in the malting barley seeds.

## 6 Conclusion and future directions

The abiotic stress adaptation and tolerance mechanisms in crop plants have become significant issues in the pursuit of global food security and sustainable agricultural practices. The Research Topic “*Abiotic Stress Adaptation and Tolerance Mechanisms in Crop Plants*” has provided a comprehensive exploration of the intricate interplay between plants and their environment, shedding light on the diverse strategies that crops employ to withstand and mitigate the detrimental effects of abiotic stresses.

In conclusion, the comprehensive exploration of abiotic stress adaptation and tolerance mechanisms in crop plants has provided valuable insights into the various strategies to cope with adverse environmental conditions. The growing challenges posed by climate change and its associated stressors demand innovative approaches to enhance crop productivity and ensure global food security. This specialty edition presents a thorough investigation into various aspects of abiotic stress response, including molecular, genetic, physiological, and metabolic processes. Through studies on transcriptomics, genomics, and metabolomics, researchers have unveiled intricate pathways and genes associated with stress tolerance. The integration of advanced techniques such as CRISPR/Cas9 gene editing has enabled targeted manipulation of stress-responsive genes, shedding light on their roles in conferring tolerance. The versatile role of polyamines as critical growth regulators in ameliorating plant abiotic stress is highlighted here. The interplay between different genes, metabolites, and pathways underscores the complexity of stress adaptation mechanisms. Moreover, studies on temperature extremes emphasize the urgency of addressing the impacts of global warming on plant growth and productivity. The research articles and reviews published in this Research Topic not only enhance our understanding of how plants respond to various stressors but also offer potential avenues for crop improvement through genetic engineering, breeding, and management practices. As the agricultural landscape evolves in the face of changing climates, these insights will guide future research and innovations aimed at developing resilient crop varieties that can thrive in adverse conditions and contribute to sustainable global food production.

### 6.1 Key insights and contributions

Throughout the Research Topic, the intricate mechanisms underlying the adaptation and tolerance of crop plants to abiotic stresses have been meticulously dissected. From the molecular and physiological responses at the cellular level to the broader implications for crop productivity and resilience, the authors have skillfully presented a holistic view of the subject. Some of the key insights that have emerged from this exploration include.

#### 6.1.1 Molecular signaling networks

This Research Topic emphasizes the pivotal role of intricate molecular signaling networks in orchestrating a plant’s response to abiotic stresses. Through a detailed analysis of stress-responsive genes, transcription factors, and signaling pathways, readers have gained a deeper understanding of how plants perceive and transmit stress signals, ultimately influencing their adaptive strategies.

#### 6.1.2 Metabolic and physiological adjustments

The Research Topic has highlighted the remarkable metabolic and physiological adjustments that plants undergo to counteract the adverse effects of abiotic stresses. From osmotic regulation to antioxidative defense mechanisms, these insights underscore the remarkable plasticity of crop plants and their capacity to reconfigure their internal systems in the face of challenges.

#### 6.1.3 Genetic variation and breeding

The exploration of genetic diversity and breeding strategies has illuminated the potential for developing stress-tolerant crop varieties. By identifying key genetic determinants and employing advanced breeding techniques, researchers and practitioners are poised to harness the power of natural variation to create resilient and high-yielding crop cultivars.

#### 6.1.4 Emerging technologies

The Research Topic has provided a glimpse into the future with its discussion of cutting-edge technologies such as genome editing, RNA interference, and omics approaches. These emerging tools offer unprecedented opportunities to engineer stress-tolerant crops with enhanced precision and efficiency.

### 6.2 Future directions

As we stand at the crossroads of scientific discovery and agricultural innovation, several advances may improve the study of abiotic stress adaptation and tolerance mechanisms in crop plants.

#### 6.2.1 Integrating multi-omics approaches

The integration of genomics, transcriptomics, proteomics, and metabolomics holds the potential to unravel the intricacies of plant stress responses with unparalleled depth. Future research should strive to integrate multi-omics data to provide a holistic view of stress adaptation mechanisms.

#### 6.2.2 Synthetic biology and engineering

The field of synthetic biology offers a unique opportunity to engineer plants with tailored stress tolerance traits. By designing and assembling novel biological components, researchers can create crops that exhibit enhanced resilience while minimizing undesirable side effects.

#### 6.2.3 Climate-adaptive crop management

With the increasing unpredictability of climatic conditions, innovative crop management strategies are needed. Future research should focus on developing site-specific, climate-adaptive approaches that optimize crop performance under diverse stress scenarios.

#### 6.2.4 Global collaboration for knowledge sharing

The challenges posed by abiotic stresses are not confined to a single region. Establishing global networks for knowledge sharing, data exchange, and collaborative research will facilitate the rapid dissemination of insights and solutions globally.

#### 6.2.5 Ethical and regulatory considerations

As we venture into the realm of genetic engineering and synthetic biology, ethical and regulatory frameworks must be established to ensure responsible innovation and the sustainable deployment of stress-tolerant crop varieties.

In conclusion, the research presented in this Research Topic has provided a profound understanding of the strategies employed by plants to thrive in challenging environments. By collectively embracing the research avenues and future directions mentioned earlier, we can work towards a sustainable and resilient agricultural future that ensures global food security.

## Author contributions

BG: Writing – review & editing, Methodology, Conceptualization, Writing – original draft. JS: Writing – review & editing.
